# Up-regulation of circRNA-0003528 promotes mycobacterium tuberculosis associated macrophage polarization via down-regulating miR-224-5p, miR-324-5p and miR-488-5p and up-regulating CTLA4

**DOI:** 10.18632/aging.104175

**Published:** 2020-12-09

**Authors:** Zikun Huang, Fangyi Yao, Jun Liu, Jianqing Xu, Yang Guo, Rigu Su, Qing Luo, Junming Li

**Affiliations:** 1 Department of Clinical Laboratory, The First Affiliated Hospital of Nanchang University, Nanchang, China

**Keywords:** tuberculosis, circRNA-0003528, CTLA4, macrophage polarization, miR-224-5p

## Abstract

Background: In this study, we selected several candidate miRNAs to study their possible relationships with tuberculosis.

Results: The expression of hsa_circ_0003528 was negatively correlated with the expression of miR-224-5p, miR-324-5p, miR-488-5p, miR-587, and miR-668, while the expression of hsa_circ_0003528 was positively correlated with the expression of miR-224-5p, miR-324-5p and miR-488-5p. No evident difference was observed between tuberculosis and healthy control groups in terms of the expression of miR-587 and miR-668.

Conclusion: The findings of this study demonstrated that miR-224-5p, miR-324-5p and miR-488-5p were all ceRNAs of circRNA-0003528 by sponging each other and CTLA4 was found to be a shared target gene of miR-224-5p, miR-324-5p and miR-488-5p. Furthermore, we found that up-regulation of circRNA-0003528 promoted tuberculosis associated macrophage polarization by promoting expression CTLA4, which was mediated by the down-regulation of miR-224-5p, miR-324-5p and miR-488-5p.

Methods: RT-qPCR and Western blot were conducted to observe the expression of hsa_circ_0003528, miRNAs and CTLA4 in different patient and cell groups to establish the potential molecular mechanisms underlying the effect of hsa_circ_0003528 on M1 to M2 macrophage polarization.

## INTRODUCTION

Tuberculosis is caused by M. TB infection, a leading condition of infectious disease attributed to significant death in the world. It is considered as a global threat to public health and has since increased its efforts in managing this disease. In fact, around 10-20 million new cases of tuberculosis are diagnosed annually, leading to over 2 millions of deaths [1].

Circular RNA (circRNA) belongs to a novel family of RNAs participating in many pathological as well as physiological processes [2, 3]. Increasing evidence has shown that multiple circRNAs function as competing endogenous RNAs (ceRNAs) to inhibit the activity of their target microRNAs (miRNAs) through binding to these target miRNAs, a process also referred to as miRNA sponging [4, 5]. Since circRNAs can generate a circular structure linked with covalent bonds, circRNAs have high stability. As a result, many circRNAs can avoid digestion by RNase in the interstitial fluid, peripheral outer blood, as well as saliva to work as molecular biomarkers in the prognosis as well as diagnosis of numerous diseases [6]. In a study by our research group to determine biomarkers of TB, 6 circRNAs were found to be overexpressed significantly in TB. These circRNAs, hsa_circ_0001953, hsa_circ_0009024, hsa_circ_0003528, hsa_circ_0008297, hsa_circ_0015879 as well as hsa_circ_0003524 also showed a close association with the severity of TB [7].

Cytotoxic T Lymphocyte-Associated Antigen 4 (CTLA-4) has been deemed an essential regulator of T cells [8]. CTLA-4 is transiently expressed on the surface of activated T cells to block T-cell function with a wide array of indirect as well as direct mechanisms [9–11]. Additionally, CTLA-4 has actually been discovered to alter T-helper (Th) cell activity toward the Th1 phenotype [12, 13]. It was hypothesized that CTLA-4Ig can transmit inhibitory signals to T cells while additionally causing the change in the M2 polarization of macrophages. As anticipated, the treatment using CTLA-4Ig reduced the expression of iNOS, an M1 marker, but substantially increased the expression of Arg1, CD206, as well as CD163, all of which are M2 markers. Moreover, the results of flow cytometry further affirmed the noticeable shift in macrophage polarization from M1 to M2. Additionally, without the treatment by CTLA-4Ig, mice given a HFD diet revealed a substantial boost in their population of CD11c-280 positive cells, whereas the mice given both a HFD diet and CTLA-4Ig showed a substantial increase in the populations of CD11c/CD206 double-positive as well as CD206 positive cells [14–16]. It was also shown that the function of CTLA426230 polymorphisms as well as the CTLA4A mutations in TB patients suggested the decreased activity of CTLA-4 in humans. As a result, TB is one of the diseases, which may explain the high incidence of the CTLA4 +6230 G phonotype in humans [16].

Distinctive states or polarization of activated macrophages were actually termed as classical M1 polarization (induced by the ligands of Toll-like receptors [TLRs] as well as interferon gamma [IFN-γ], with signaling conducted by STAT1) and alternative M2 polarization (induced by interleukin-13 [IL-13], interleukin-4 [IL-4] as well as interleukin-10 [IL-10] via STAT6 and STAT3, respectively) [17–19]. The M1 proinflammatory polarization is featured by a high level of expression of proinflammatory cytokines such as tumor necrosis factor alpha [TNF-α] as well as high levels of reactive nitrogen and Th1 responses, which result in strong solid tumoricidal as well as microbicidal activities [18, 20]. The M2 anti-inflammatory polarization is featured by high levels of expression of anti-inflammatory cytokines such as IL-10, which result in the promotion of tissue remodeling and recovery [20, 21]. Mattila et al discovered that significant levels of proinflammatory as well as anti-inflammatory macrophages are present together in the lungs of TB patients, indicating that the TB infection can regulate macrophage polarization [22–24]. Some other studies also confirmed that during the onset of TB, macrophages are mainly undergoing M1 polarization [25–27].

Our previous study has identified some circular RNAs that are differentially expressed in TB [7]. It was also shown that CTLA4 was reported to be involved in the pathogenesis of TB and macrophage polarization [28, 29]. In this study, we attempted to identify the candidate circRNA as the biomarker of TB and explored the downstream signaling pathway of the studied circRNA by using computational in-silico analysis and functional analysis.

## RESULTS

### Plasma hsa_circ_0003528 was elevated in TB patients

In our previous study, we utilized a circRNA microarray to analyze plasma hsa_circ_0003528 in patients with active pulmonary TB in comparison with that in healthy controls. The microarray data were subsequently validated by RT-qPCR of the study population, which included 170 TB patients, 40 pneumonia patients, 40 COPD patients, 40 lung cancer patients, and 150 healthy controls. The clinical characteristics of the study population were presented in the previous literature [25, 26]. Moreover, the validation stage presented good consistency between microarray and RT-qPCR data [25, 26], indicating a significant elevation of hsa_circ_0003528 in TB patients.

### Correlation between miRNAs expression and hsa_circ_0003528 expression

**Figure 1:**
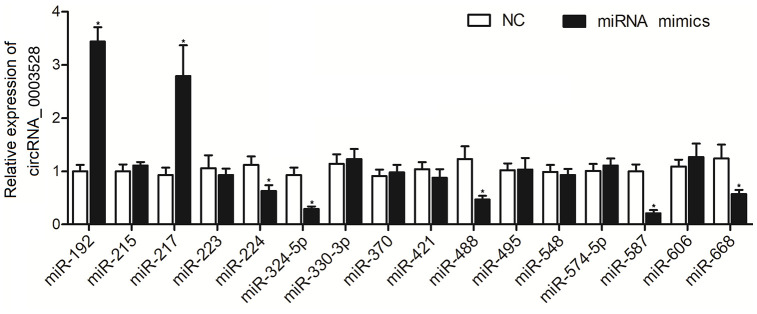
Among the 16 candidate miRNAs, the expressions of miR-224-5p, miR-324-5p, miR-488-5p, miR-587, and miR-668 were negatively correlated with the expression of hsa_circ_0003528, and the expressions of miR-192 and miR-217 were positively correlated with the expression of hsa_circ_0003528 (*P value < 0.05 vs. NC group).

To explore the possible miRNAs sponged by hsa_circ_0003528, a computational analysis was performed. As shown in Figure 1, 16 candidate miRNAs were identified, and the according miRNA mimics were transfected into THP-1 cells to observe their effects on the expression of hsa_circ_0003528. Accordingly, the RT-qPCR results presented an evident negative correlation between expression of hsa_circ_0003528 and expression of miR-224-5p, miR-324-5p, miR-488-5p, miR-587, and miR-668, and a significant positive correlation between expression of hsa_circ_0003528 and miR-192 and miR-217.

### MiR-224-5p, miR-324-5p and miR-488-5p were down-regulated in TB patients

**Figure 2:**
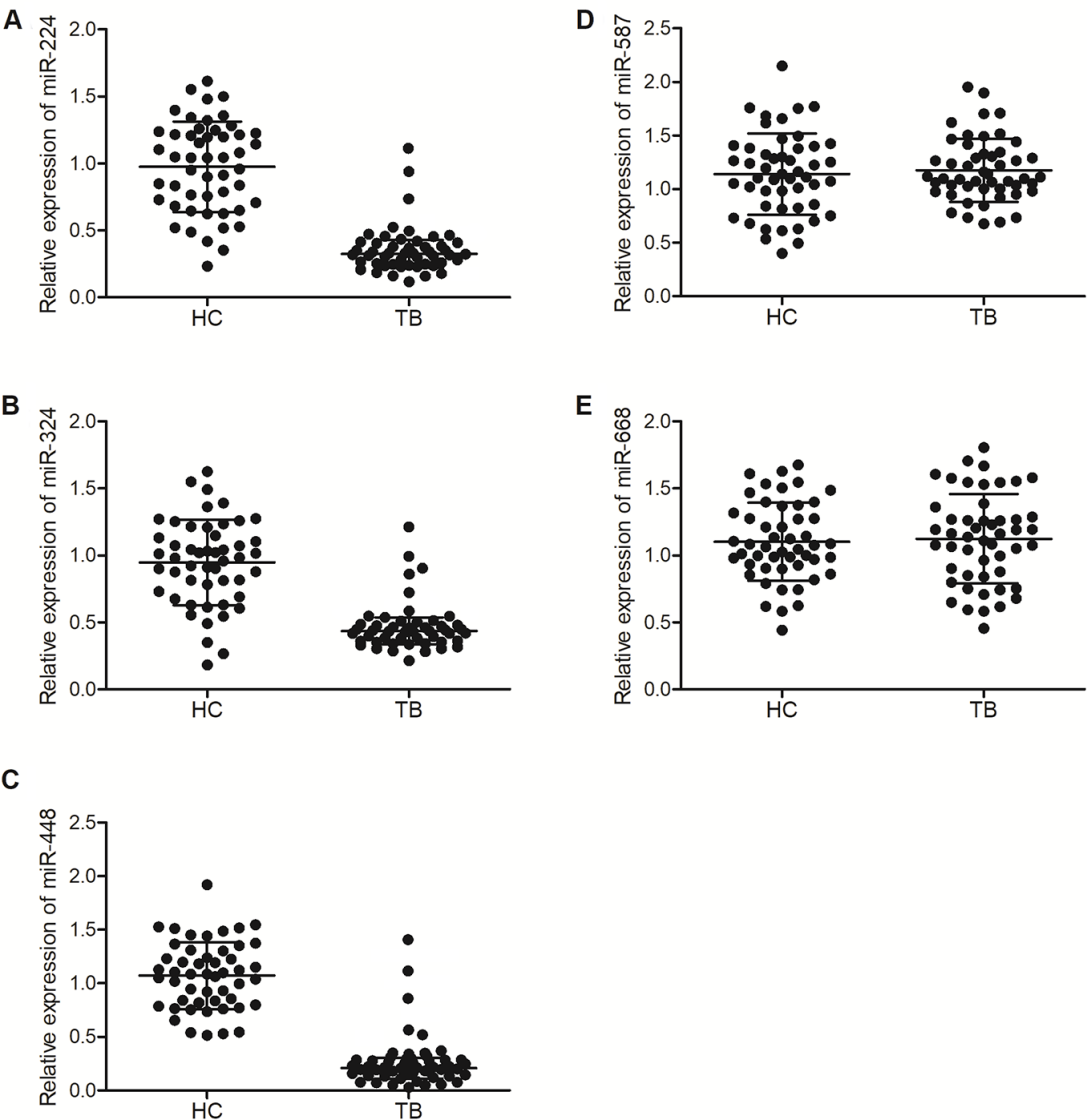
**MiR-224-5p, miR-324-5p and miR-488-5p were down-regulated in TB patients.** (**A**) Relative expression of miR-224-5p in TB patients and healthy controls; (**B**) Relative expression of miR-324-5p in TB patients and healthy controls; (**C**) Relative expression of miR-488-5p in TB patients and healthy controls; (**D**) Relative expression of miR-587 in TB patients and healthy controls; (**E**) Relative expression of miR-668 in TB patients and healthy controls.

In our previous study, to validate the microarray profile of hsa_circ_0003528, 50 patients with active pulmonary TB (as the TB group) and 50 healthy controls (as the HC group) were recruited. In this study, we further validated the correlations between the expression of miRNAs (including miR-224-5p, miR-324-5p, miR-488-5p, miR-587 and miR-668) and the expression of hsa_circ_0003528 among these patients. As shown in Figure 2, the relative expression of miR-224-5p (Figure 2A), miR-324-5p (Figure 2B) and miR-488-5p (Figure 2C) was all markedly down-regulated in the TB group, while no evident difference was observed between the TB and HC groups in terms of the expression of miR-587 (Figure 2D) and miR-668 (Figure 2E).

### Hsa_circ_0003528 functioned as a sponge of miR-324-5p, miR-224-5p and miR-488-5p

**Figure 3:**
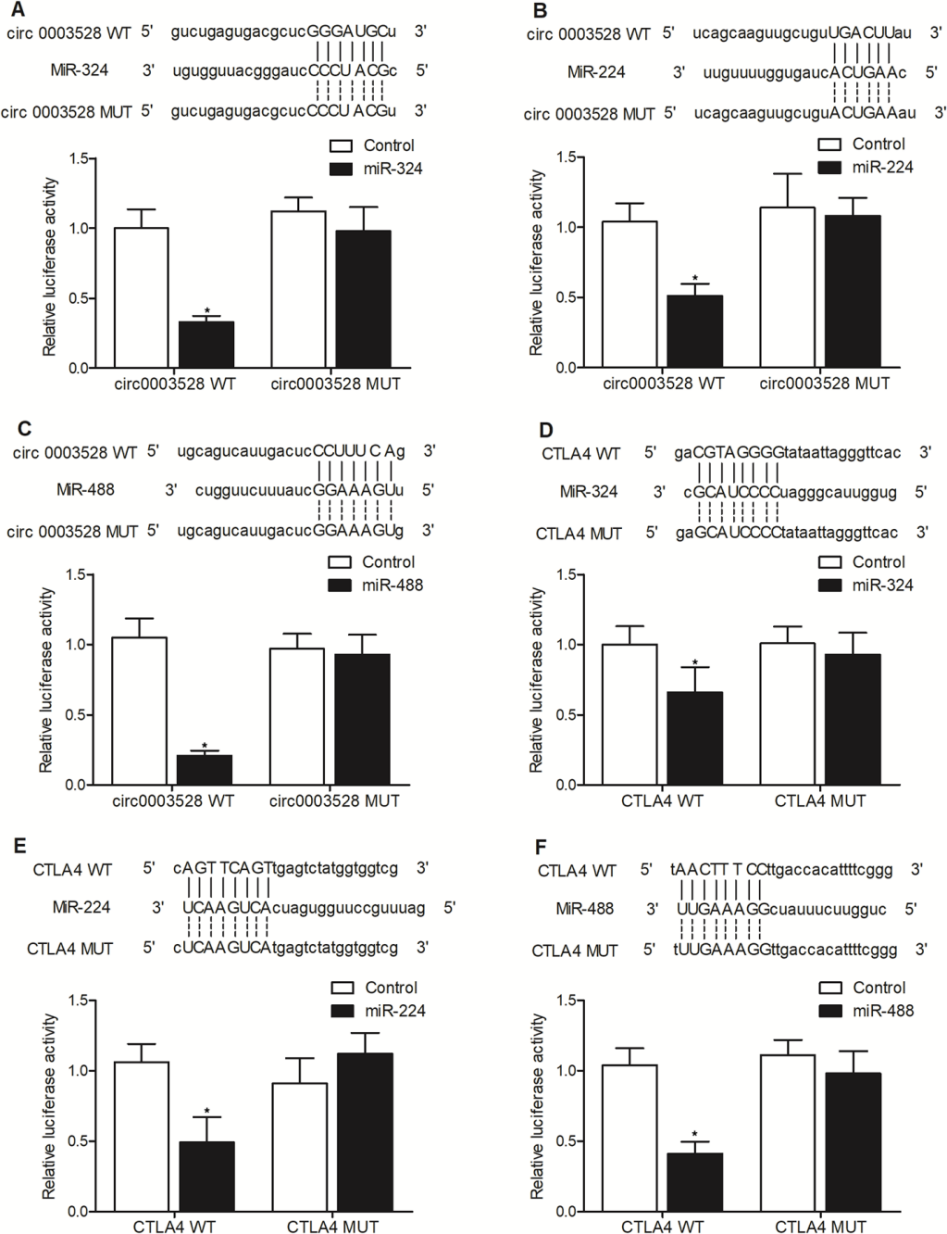
Hsa_circ_0003528 functioned as a sponge of miR-324-5p, miR-224-5p and miR-488-5p, while CTLA4 mRNA was targeted by miR-224-5p, miR-324-5p and miR-488-5p (*P value < 0.05 vs. control group). (**A**) Computational analysis and luciferase assay of the molecular relationship between hsa_circ_0003528 and miR-324-5p; (**B**) Computational analysis and luciferase assay of the molecular relationship between hsa_circ_0003528 and miR-224-5p; (**C**) Computational analysis and luciferase assay of the molecular relationship between hsa_circ_0003528 and miR-488-5p; (**D**) Computational analysis and luciferase assay of the molecular relationship between CLTA4 mRNA and miR-324-5p; (**E**) Computational analysis and luciferase assay of the molecular relationship between CLTA4 mRNA and miR-224-5p; (**F**) Computational analysis and luciferase assay of the molecular relationship between CLTA4 mRNA and miR-488-5p.

To study the molecular relationship between hsa_circ_0003528 and miR-224-5p, miR-324-5p and miR-488-5p, luciferase assays were conducted to confirm the specific binding between hsa_circ_0003528 and above miRNAs. Different putative binding sites of hsa_circ_0003528 were located on miR-324-5p (Figure 3A), miR-224-5p (Figure 3B) and miR-488-5p (Figure 3C), and the luciferase activity of wild-type hsa_circ_0003528 was evidently suppressed in THP-1 cells transfected with miR-324-5p (Figure 3A), miR-224-5p (Figure 3B) and miR-488-5p (Figure 3C). Therefore, it can be validated that miRNAs including miR-224-5p, miR-324-5p and miR-488-5p could bind to hsa_circ_0003528.

### CTLA4 mRNA was targeted by miR-224-5p, miR-324-5p and miR-488-5p

The computational analysis also identified putative binding sites of miR-324-5p (Figure 3D), miR-324-5p (Figure 3E) and miR-488-5p (Figure 3F), respectively, on the 3’UTR of CTLA4 mRNA. The luciferase assay indicated that the expression of wild-type CTLA4 mRNA was markedly decreased in THP-1 cells transfected with miR-324-5p (Figure 3D), miR-224-5p (Figure 3E) and miR-488-5p (Figure 3F). Therefore, it can be validated that CTLA4 mRNA was targeted by miR-224-5p, miR-324-5p and miR-488-5p.

### Establishment of a molecular signaling pathway

**Figure 4:**
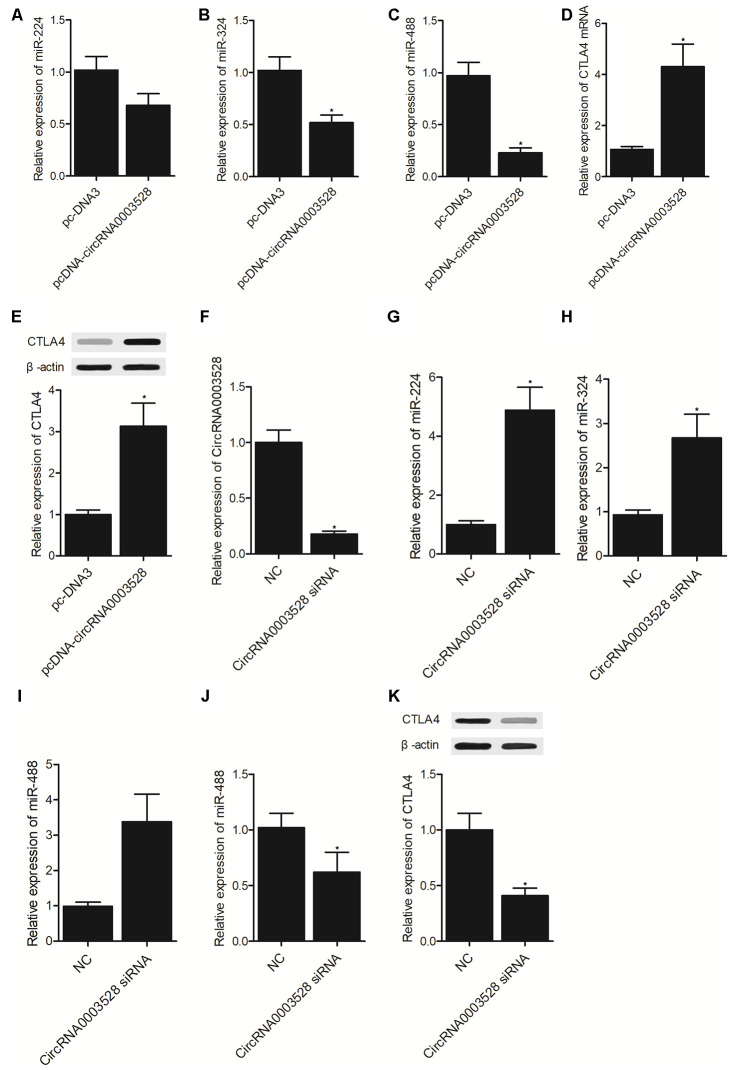
Establishment of a molecular signaling pathway (*P value < 0.05 vs. NC group). (**A**) The expression of miR-224-5p in THP-1 cells transfected with hsa_circ_0003528 and the empty control; (**B**) The expression of miR-324-5p in THP-1 cells transfected with hsa_circ_0003528 and the empty control; (**C**) The expression of miR-488-5p in THP-1 cells transfected with hsa_circ_0003528 and the empty control; (**D**) The expression of CTLA4 mRNA in THP-1 cells transfected with hsa_circ_0003528 and the empty control; (**E**) The expression of CTLA4 protein in THP-1 cells transfected with hsa_circ_0003528 and the empty control; (**F**) The expression of hsa_circ_0003528 in U937 cells transfected with hsa_circ_0003528 siRNA and the negative control; (**G**) The expression of miR-224-5p in U937 cells transfected with hsa_circ_0003528 siRNA and the negative control; (**H**) The expression of miR-324-5p in U937 cells transfected with hsa_circ_0003528 siRNA and the negative control; (**I**) The expression of miR-488-5p in U937 cells transfected with hsa_circ_0003528 siRNA and the negative control; (**J**) The expression of CTLA4 mRNA in U937 cells transfected with hsa_circ_0003528 siRNA and the negative control; (**K**) The expression of CTLA4 protein in U937 cells transfected with hsa_circ_0003528 siRNA and the negative control.

THP-1 cells were transfected with hsa_circ_0003528 or an empty vector. It was found that the expression of miR-224-5p (Figure 4A), miR-324-5p (Figure 4B) and miR-488-5p (Figure 4C) was all suppressed while the expression of CTLA4 mRNA (Figure 4D) and protein (Figure 4E) was promoted by the transfection of hsa_circ_0003528 in THP-1 cells. Furthermore, U937 cells were transfected with hsa_circ_0003528 siRNA and a negative control (Figure 4F), and the expression of miR-224-5p (Figure 4G), miR-324-5p (Figure 4H) and miR-488-5p (Figure 4I) was all up-regulated while the expression of CTLA4 mRNA (Figure 4J) and protein (Figure 4K) was down-regulated by the transfection of hsa_circ_0003528 siRNA in U937 cells.

### The hsa_circ_0003528 signaling pathway was associated with macrophage polarization induced by IFN-γ + LPS or IL-4

**Figure 5:**
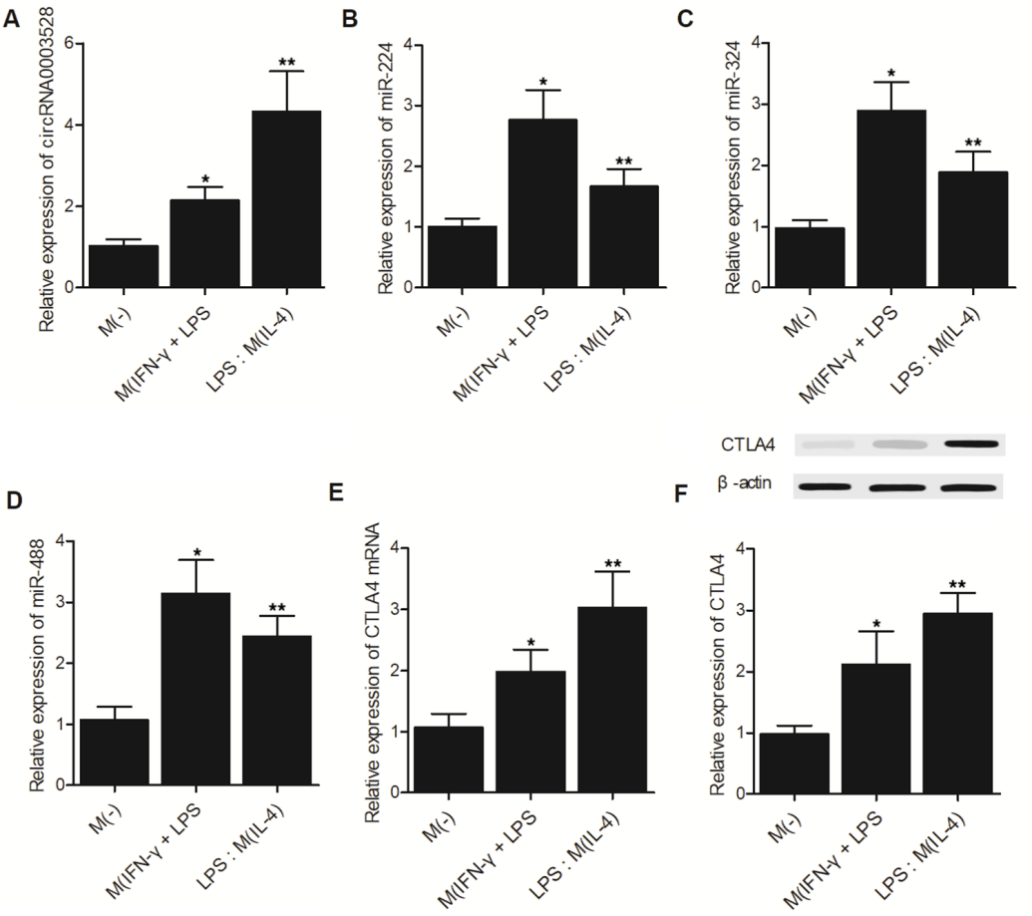
The hsa_circ_0003528 signaling pathway was associated with macrophage polarization induced by IFN-γ + LPS or IL-4 (*P value < 0.05 vs. M (-) group; ** P value < 0.05 vs. M(IFN-γ + LPS) group). (**A**) The expression of hsa_circ_0003528 in the M(-) group, M(IFN-γ + LPS) group and M(IL-4) group; (**B**) The expression of miR-224-5p in the M(-) group, M(IFN-γ + LPS) group and M(IL-4) group; (**C**) The expression of miR-324-5p in the M(-) group, M(IFN-γ + LPS) group and M(IL-4) group; (**D**) The expression of miR-488-5p in the M(-) group, M(IFN-γ + LPS) group and M(IL-4) group; (**E**) The expression of CTLA4 mRNA in the M(-) group, M(IFN-γ + LPS) group and M(IL-4) group. (**F**) The expression of CTLA4 protein in the M(-) group, M(IFN-γ + LPS) group and M(IL-4) group.

THP-1 cells were grouped as the M(-) group, M(IFN-γ + LPS) group and M(IL-4) group. The expression of hsa_circ_0003528 (Figure 5A) and CTLA4 mRNA (Figure 5E)/protein (Figure 5F) was increased in cells undergoing macrophage polarization induced by IFN-γ + LPS or IL-4. However, although the expression of miR-224-5p (Figure 5B), miR-324-5p (Figure 5C) and miR-488-5p (Figure 5D) was increased in macrophages treated with IFN-γ + LPS, the M(IL-4) group showed reduced expression of these miRNAs.

### The hsa_circ_0003528 signaling pathway was associated with M1 to M2 macrophage polarization

**Figure 6:**
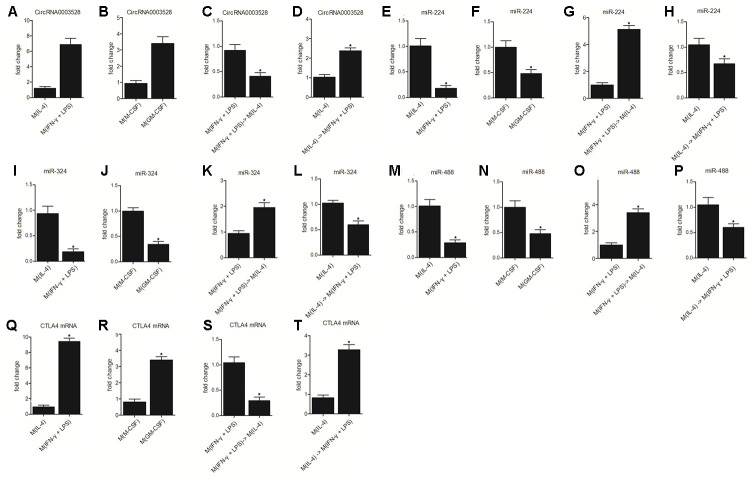
The hsa_circ_0003528 signaling pathway was associated with M1 to M2 macrophage polarization (* P value < 0.05 vs. M(IL-4) group). (**A**) The expression of hsa_circ_0003528 between the M(IL-4) group and M(IFN-γ + LPS) group; (**B**) The expression of hsa_circ_0003528 between the M(M-CSF) group and M(GM-CSF) group; (**C**) The expression of hsa_circ_0003528 among the M(IFN-γ + LPS) group, M(IFN-γ + LPS) group and M(IL-4) group; (**D**) The expression of hsa_circ_0003528 among the M(IL-4) group, M(IL-4) group and M(IFN-γ + LPS) group. (**E**) The expression of miR-224-5p between the M(IL-4) group and M(IFN-γ + LPS) group; (**F**) The expression of miR-224-5p between the M(M-CSF) group and M(GM-CSF) group; (**G**) The expression of miR-224-5p among the M(IFN-γ + LPS) group, M(IFN-γ + LPS) group and M(IL-4) group; (**H**) The expression of miR-224-5p among the M(IL-4) group, M(IL-4) group and M(IFN-γ + LPS) group. (**I**) The expression of miR-324-5p between the M(IL-4) group and M(IFN-γ + LPS) group; (**J**) The expression of miR-324-5p between the M(M-CSF) group and M(GM-CSF) group; (**K**) The expression of miR-324-5p among the M(IFN-γ + LPS) group, M(IFN-γ + LPS) group and M(IL-4) group; (**L**) The expression of miR-324-5p among the M(IL-4) group, M(IL-4) group, and M(IFN-γ + LPS) group. (**M**) The expression of miR-488-5p between the M(IL-4) group and M(IFN-γ + LPS) group; (**N**) The expression of miR-488-5p between the M(M-CSF) group and M(GM-CSF) group; (**O**) The expression of miR-488-5p among the M(IFN-γ + LPS) group, M(IFN-γ + LPS) group, and M(IL-4) group; (**P**) The expression of miR-488-5p among the M(IL-4) group, M(IL-4) group, and M(IFN-γ + LPS) group. (**Q**) The expression of CTLA4 mRNA between the M(IL-4) group and M(IFN-γ + LPS) group; (**R**) The expression of CTLA4 mRNA between the M(M-CSF) group and M(GM-CSF) group; (**S**) The expression of CTLA4 mRNA among the M(IFN-γ + LPS) group, M(IFN-γ + LPS) group and M(IL-4) group; (**T**) The expression of CTLA4 mRNA among the M(IL-4) group, M(IL-4) group and M(IFN-γ + LPS) group.

The activity of the hsa_circ_0003528 signaling pathway was further compared between the M(IL-4) group, M(IFN-γ + LPS) group, M(M-CSF) group, M(GM-CSF) group, M(IFN-γ + LPS) group, M(IFN-γ + LPS), M(IL-4) group, M(IL-4) group, M(IL-4) group and M(IFN-γ + LPS) group. Accordingly, the expression of hsa_circ_0003528 (Figure 6A and 6B) and CTLA4 mRNA (Figure 6Q and 6R) was increased in the M(IFN-γ + LPS) group and M(GM-CSF) group compared with that in the M(IL-4) group (Figure 6A and 6Q) and M(M-CSF) group (Figure 6B and 6R), respectively. The change in macrophage polarization also reduced the expression of hsa_circ_0003528 (Figure 6C and 6D) and CTLA4 mRNA (Figure 6S and 6T), i.e. the treatment with IL-4 reversed increased expression of hsa_circ_0003528 and CTLA4 mRNA caused by IFN- γ + LPS (Figure 6C and 6D) and the treatment with IFN- γ + LPS reversed increased expression of hsa_circ_0003528 and CTLA4 mRNA caused by IL-4 (Figure 6S and 6T). In addition, we also evaluated the expression of the expression of miR-224-5p (Figure 6E–6H), miR-324-5p (Figure 6I–6L) and miR-488-5p (Figure 6M–6P) in each treatment group. In line with the result about hsa_circ_0003528 (Figure 6A and 6B) and CTLA4 mRNA in each group, it was found that miR-224-5p (Figure 6E–6H), miR-324-5p (Figure 6I–6L) and miR-488-5p (Figure 6M–6P) showed opposite tendency against that of hsa_circ_0003528 and CTLA4 mRNA among these treatment groups.

### Overexpression of hsa_circ_0003528 in cells of macrophages polarization induced by IFN-γ + LPS regulated the expression of chemokines

**Figure 7:**
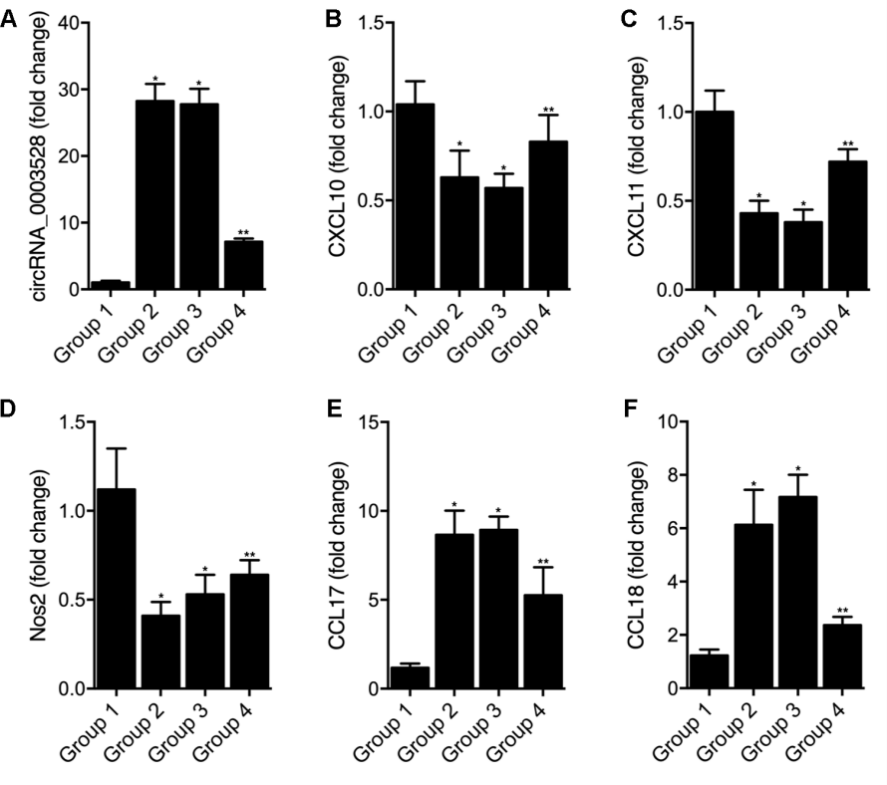
Effect of overexpression of hsa_circ_0003528 on cells undergoing macrophages polarization induced by IFN-γ + LPS (Group 1: M(IFN-γ + LPS), pcDNA; Group 2: M(IFN-γ + LPS), pcDNA-circ_0003528; Group 3: M(IFN-γ + LPS), pcDNA-circ_0003528, NC siRNA; Group 4: M(IFN-γ + LPS), pcDNA-circ_0003528, circ_0003528 siRNA; * P value < 0.05 vs. Group 1; ** P value < 0.05 vs. Group 3). (**A**) Fold change of circ_0003528 expression in four THP-1 cell groups; (**B**) Fold change of CLCL10 expression in four THP-1 cell groups; (**C**) Fold change of CLCL11 expression in four THP-1 cell groups; (**D**) Fold change of Nos2 expression in four THP-1 cell groups; (**E**) Fold change of CCL17 expression in four THP-1 cell groups; (**F**) Fold change of CCL18 expression in four THP-1 cell groups.

**Figure 8:**
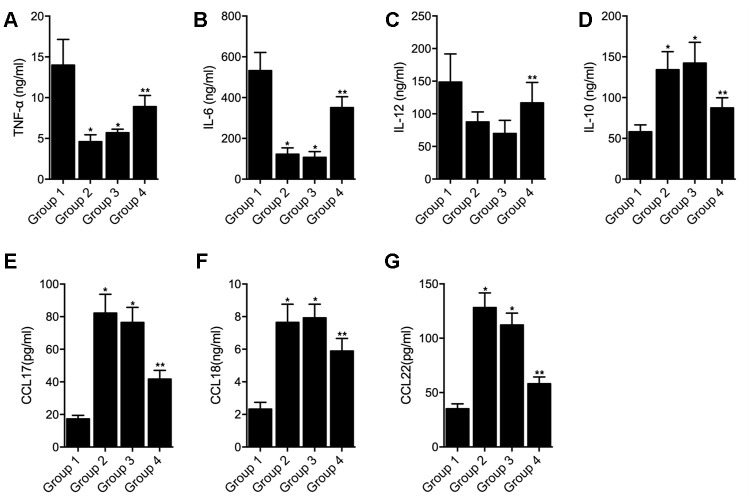
Effect of overexpression of hsa_circ_0003528 on production of inflammatory cytokines induced by IFN-γ + LPS (Group 1: M(IFN-γ + LPS), pcDNA; Group 2: M(IFN-γ + LPS), pcDNA-circ_0003528; Group 3: M(IFN-γ + LPS), pcDNA-circ_0003528, NC siRNA; Group 4: M(IFN-γ + LPS), pcDNA-circ_0003528, circ_0003528 siRNA; * P value < 0.05 vs. Group 1; ** P value < 0.05 vs. Group 3). (**A**) Concentration of TNF-α in four THP-1 cell groups; (**B**) Concentration of IL-6 in four THP-1 cell groups; (**C**) Concentration of IL-12 in four THP-1 cell groups; (**D**) Concentration of IL-10 in four THP-1 cell groups; (**E**) Concentration of CCL17 in four THP-1 cell groups; (**F**) Concentration of CCL18 in four THP-1 cell groups; (**G**) Concentration of CCL22 in four THP-1 cell groups.

THP-1 cells undergoing macrophages polarization induced by IFN-γ + LPS were transfected with empty vector (as Group 1), hsa_circ_0003528 (as Group 2), NC siRNA (as Group 3) or circ_0003528 siRNA (as Group 4). As shown in Figure 7, the successful transfection of circ_0003528 or circ_0003528 siRNA were validated by the varied expression of circ_0003528 (Figure 7A). And the expression of CLCL10 (Figure 7B), CXCl11 (Figure 7C) and Nos2 (Figure 7D) was decreased while the expression of CCL17 (Figure 7E) and CCL18(Figure 7F) was increased by the over-expression of hsa_circ_0003528. The concentrations of TNF-α (Figure 8A), IL-6 (Figure 8B) and IL-12 (Figure 8C) were down-regulated while the concentrations of IL-10 (Figure 8D), CCL17 (Figure 8E), CCL18 (Figure 8F) and CCL22 (Figure 8G) were up-regulated in THP-1 cells undergoing macrophages polarization induced by IFN-γ + LPS that were transfected with hsa_circ_0003528. Also, although the co-transfection of NC siRNA and circ_0003528 exhibited no evident effect upon the expression of the above investigated parameters, when replacing NC siRNA with circ_0003528 siRNA, the dysregulated expressions of CLCL10 (Figure 7B), CXCl11 (Figure 7C), Nos2 (Figure 7D), CCL17 (Figure 7E), CCL18(Figure 7F), TNF-α (Figure 8A), IL-6 (Figure 8B) and IL-12 (Figure 8C), IL-10 (Figure 8D), CCL17 (Figure 8E), CCL18 (Figure 8F) and CCL22 (Figure 8G) was all partly recovered by the presence circ_0003528 siRNA.

## DISCUSSION

In a recent study, it was found that that levels of hsa_circ_0001953, hsa_circ_0009024, hsa_circ_0003528, hsa_circ_0008297, hsa_circ_0015879 as well as hsa_circ_0003524 were increased remarkably in patients with TB [7]. Another research analyzed the characteristics of miRNA expression during the onset (30 days) as well as advanced (60 days) stages of TB in an experimental model. The results displayed the expression of a total of 598 miRNAs, including 37 miRNAs whose expression was remarkably changed during the early stage of TB infection (9 miRNAs whose expression was remarkably down-regulated as well as 18 miRNAs whose expression was remarkably up-regulated), whereas the expression of 12 miRNAs was greatly changed during the advanced stage of TB, suggesting that different miRNAs were differently expressed during different stage of TB [30]. With an attempt to explore the downstream signaling pathway of the differentially expressed circRNAs, we performed computational analysis to identify the potential ceRNAs, i.e. the miRNAs that possess potential binding sites and furthermore found out the potential target genes of those candidate miRNAs and as a final step, we collectively analyzed in a comprehensive way by combining the in-silico analysis and searching literature. Those target genes that are related to the control of TB associated macrophage polarization. As hsa_circ_0003528 was found to have most multiple sponging miRNAs with target gene of interest, the present study is focused on hsa_circ_0003528. To test the interaction between the candidate miRNAs and hsa_circ_0003528, we transfected the mimics of those candidate miRNAs into cultured cells to evaluate their effect on the expression of hsa_circ_0003528 and found that the expression of hsa_circ_0003528 was decreased in the presence of miR-224-5p, miR-324-5p, miR-488-5p, miR-587, and miR-668, while the transfection of miR-192 and miR-217 elevated the expression of hsa_circ_0003528. Similarly, another research presented that the expression of 15 miRNAs (such as miR-142-5p, miR-29b, as well as miR-21) was actually up-regulated while the expression of 14 miRNAs (such as miR-324-5p) was down-regulated in patients with TB [31]. The downregulation of miR-324-5p in this reference was in line with our result regarding the change of miR-324-5p in the pathogenesis of TB. Another recent research detected 1223 miRNAs in the serum of patients with TB and presented that the expression of 92 miRNAs was altered significantly, including 59 miRNAs whose expression was remarkably up-regulated and 33 miRNAs including miR-488-5p whose expression was remarkably down-regulated [32] and this result is consistent with the result of our study regarding the change of miR-488-5p.

Furthermore, multiple miRNAs have been reported to regulate the macrophage polarization. For example, the evaluation of miRNA expression during macrophage polarization indicated that the expression of 76 miRNAs, such as miR-146a, miR-155-5p as well as other miRNAs considered as the markers of M1 polarization, was up-regulated [33]. In addition, the transfection of macrophages with miR-488-5p minimized the roundness of macrophages while inhibiting the expression of inflammatory markers on the transcriptional [34]. Macrophage polarization has been reported to be involved in the pathogenesis of TB as well as other inflammation and infection process [17–19]. The macrophage polarization was characterized with two phases, M1 and M2 [18]. The M1 phase is characterized with an activated inflammatory response showing an elevated expression and production of proinflammatory cytokines including but not limited to tumor necrosis factor alpha [TNF-α] as well as high levels of reactive nitrogen and Th1 responses [18, 20]. However, the M2 phase was featured as an anti-inflammatory response by increasing the expression and production of anti-inflammatory cytokines such as IL-10 [20, 21]. It has been shown that dysregulation of macrophage polarization has been found to be involved in the development of TB [22–24]. Especially, an over-activated inflammatory response caused by over-driven M1 phase of macrophage polarization is responsible for the initiation of TB infection [25–27].

In this study, we enrolled subjects with or without TB and compared their expression of candidate miRNAs. We found that the expression of miR-224-5p, miR-324-5p and miR-488-5p were all markedly down-regulated in the TB group compared with that in the HC group, while no evident difference was observed between TB and HC groups in terms of the expression of miR-587 and miR-668. The relative expression of miR-224-5p, miR-324-5p and miR-488-5p was the lowest and the relative expression of hsa_circ_0003528 was the highest in the TB group. Furthermore, we found that Hsa_circ_0003528 functioned as a sponge of miR-324-5p, miR-224-5p and miR-488-5p, while CTLA4 mRNA was targeted by miR-224-5p, miR-324-5p and miR-488-5p. The expression of miR-224-5p, miR-324-5p and miR-488-5p were all suppressed while the expression of CTLA4 mRNA and protein was promoted by the transfection of hsa_circ_0003528.

As a receptor in the CD28 family, CTLA4 blocks the proliferation of T cells via binding to B7 molecules [35]. Furthermore, the +49 A > G (rs231775) as well as +6230 (rs3087243) single nucleotide polymorphisms (SNPs) in CTLA4 were revealed to cause potent linkage disequilibrium [36–38]. In addition, healthy controls and TB patients showed no remarkable variations in their CTLA4 11430 as well as +6230 frequencies, although the +49 genotype provided a protective effect against COPD [39]. In addition, another study on Africans showed that the +6230 G mutation notably impacted the severity of TB as well as the number and size of pulmonary cavities [16]. Additionally, it was found that the lysate of L. acidophilus promoted the anti-tumor effects of CTLA-4 by reducing the quantity of Treg as well as M2 cells, while increasing the levels of T cells and IFN-γ, Granzyme B, and TNF-α [29]. In this study, we found that the hsa_circ_0003528 signaling pathway was associated with macrophage polarization induced by IFN-γ + LPS or IL-4, and the M1 to M2 macrophage polarization. Moreover, the overexpression of hsa_circ_0003528 in cells undergoing macrophages polarization induced by IFN-γ + LPS regulated the expression of chemokines.

However, there are two major limitations of our study. Apart from the study upon patients, the lack of *in vivo* experiment with appropriate animal models limited the validation of the conclusions drawn from our study. Also, for the patient sample collection, the sample size was also relatively small which is also a limitation of our study. In our future study, apart from the necessity to introduce an appropriate animal model, larger sample size is also preferred.

## CONCLUSIONS

The findings of this study demonstrated that miR-224-5p, miR-324-5p and miR-488-5p were all ceRNAs of circRNA-0003528 by sponging each other and CTLA4 was found to be a shared target gene of miR-224-5p, miR-324-5p and miR-488-5p. Furthermore, we found that up-regulation of circRNA-0003528 could promote TB associated macrophage polarization by up-regulating the expression of CTLA4.

## MATERIALS AND METHODS

### Patient recruitment

In this study, we first included 50 patients with active pulmonary TB (as the TB group) and 50 healthy controls (as the HC group). We further validated the correlations between the expression of miRNAs (including miR-224-5p, miR-324-5p, miR-488-5p, miR-587 and miR-668) and the expression of hsa_circ_0003528 among these subjects. The clinical characteristics of the study population were summarized and compared among different groups using Student’s t tests. This study was performed based on the Declaration of Helsinki and Chinese GCP and GLP. Written informed consent forms were acquired from all subjects before the study was started. The entire study protocol was approved by the Ethical Committee of our hospital.

### Cell culture, polarization and transfection

THP-1 and U937 cells were cultured in RPMI 1640 medium containing 10% FBS and 1.75μL/500 mL β-mercaptoethanol (Sigma) and differentiated into macrophages by treatment with 5ng/mL phorbol-12-myristate-13-acetate (PMA) (Sigma) overnight.

In cellular experiment 1, the THP-1 and U937 cells were transfected with either scramble control or the candidate miRNA mimics to compare their effect on the expression of circRNA_0003528. Those candidate miRNAs include miR-192, miR-215, miR-217, miR-223, miR-224-5p, miR-324-5p, miR-330-3p, miR-370, miR-421, miR-488-5p, miR-495, miR-548, miR-574-5p, miR-587, miR-606, miR-668.

In cellular experiment 2, the THP-1 and U937 cells were divided into 2 groups, i.e., 1. pcDNA group (THP-1 and U937 cells transfected with an empty vector); and 2. pcDNA-hsa_circ_0003528 group (THP-1 and U937 cells transfected with the vector carrying hsa_circ_0003528).

In cellular experiment 3, the THP-1 and U937 cells were divided into 2 groups, i.e., 1. NC group (THP-1 and U937 cells transfected with a scramble siRNA); and 2. hsa_circ_0003528 siRNA group (THP-1 and U937 cells transfected with hsa_circ_0003528 siRNA).

In cellular experiment 4, the THP-1 and U937 cells were divided into 3 groups, i.e., 1. M (-) group (no macrophage polarization control group); 2. M (IFN-γ + LPS) group (cells were treated with 20 ng/ml IFN-γ (R&D systems, USA) + 100 ng/mL of LPS (sigma) for 18 hours); and 3. M (IL-4) group (cells were treated with 20 ng/mL of IL-4 (PeproTech, USA) for 18 hours).

In cellular experiment 5, the THP-1 and U937 cells were divided into 2 groups, i.e., 1. M (M-CSF) group (control group); and 2. M (GM-CSF) group (cells were treated with 20ng/mL GM-CSF for 18 hours).

In cellular experiment 6, the THP-1 and U937 cells were divided into 2 groups, i.e., 1. M (IFN-γ + LPS) group (cells were treated with 20 ng/ml IFN-γ + 100 ng/mL of LPS for 18 hours); and 2. M (IFN-γ + LPS) to M (IL-4) group (cells in groups were subsequently treated with 20 ng/mL of IL-4 for another 18 hours).

In cellular experiment 7, the THP-1 and U937 cells were divided into 2 groups, i.e., 1. M (IL-4) group (cells treated with 20 ng/mL of IL-4 for 18 hours); and 2. M (IL-4) to M (IFN-γ + LPS) group (cells in groups were subsequently treated with 20 ng/ml IFN-γ + 100 ng/mL of LPS for another 18 hours). The treatment was described as previously [25, 26].

In cellular experiment 8, the THP-1 and U937 cells were divided into 4 groups, i.e., 1. M (IFN-γ + LPS) + pcDNA group (cells treated with 20 ng/mL of IL-4 for 18 hours as well as transfection with empty vector as control); 2. M (IFN-γ + LPS) + pcDNA-circ_0003528 group (cells treated with 20 ng/mL of IL-4 for 18 hours as well as transfection with vector containing hsa_circ_0003528); 3. M (IFN-γ + LPS) + pcDNA-circ_0003528, NC siRNA group (cells treated with 20 ng/mL of IL-4 for 18 hours as well as transfection with vector containing hsa_circ_0003528 and NC siRNA); 2. M (IFN-γ + LPS) + pcDNA-circ_0003528, circ_0003528 siRNA group (cells treated with 20 ng/mL of IL-4 for 18 hours as well as transfection with vector containing hsa_circ_0003528 and circ_0003528 siRNA).

All transfection was done using the Lipofectamine ^2000^ reagent (Invitrogen, Carlsbad, CA) in accordance with the standard protocol provided on the product manual of the manufacturer. The transfected cells were harvested 48 h after transfection to assay the expression of target genes.

### Target prediction

The candidate circRNAs in our study were selected by utilization of Circular RNA Interactome (https://circinteractome.irp.nia.nih.gov/) and the candidate miRNAs in our study were selected by utilization of some frequently used target gene prediction databases including miRDB and TargetScan.

### RNA isolation and real-time PCR

Real-time PCR was done to assay the expression of hsa_circ_0003528, miR-224-5p, miR-324-5p, miR-488-5p, miR-587, miR-668, and CTLA4 mRNA in each sample. In brief, the collected samples were subject to the treatment with a QIAzol Lysis Reagent (Qiagen, Germantown, MD) and subsequent RNA extraction operations carried out using a miRNeasy Mini assay kit (Qiagen, Germantown, MD) or a miRCURY RNA Isolation kit (Qiagen, Germantown, MD) in accordance with the standard protocol provided on the product manual of the manufacturer to isolate total RNA content in each sample for miRNA and mRNA amplification, respectively. Then, the isolated total RNA samples were reverse transcribed into cDNA templates by using a Universal cDNA Synthesis II assay kit (Qiagen, Germantown, MD) in accordance with the standard protocol provided on the product manual of the manufacturer. Finally, in the step of real time PCR, the reactions were carried out using an ExiLENT SYBR Green Master Mix reagent (Qiagen, Germantown, MD) and microRNA LNA PCR primers (Qiagen, Germantown, MD) on a Light Cycler 480 real time PCR machine (Roche, Nutley, NJ) in accordance with the standard protocol provided on the product manual of the manufacturer. The relative expression of hsa_circ_0003528 (Forward primer: 5’-GTAACCAGCAGCCTGGACTC-3; Reverse primer: 5’-GCAACTTGCTGACCAGAACA-3’), miR-224-5p (Forward primer: 5’-CAAGTCACTAGTGGTTCC-3’; Reverse primer: 5’-GAACATGTCTGCGTATCTC-3’), miR-324-5p (Forward primer: 5’- CATCCCCTAGGGCATTG-3’; Reverse primer: 5’-GAACATGTCTGCGTATCTC-3’), miR-488-5p (Forward primer: 5’-CCCAGATAATGGCACTC-3’; Reverse primer: 5’-GAACATGTCTGCGTATCTC-3’), miR-587 (Forward primer: 5’-TTCCATAGGTGATGAGTC-3’; Reverse primer: 5’-GAACATGTCTGCGTATCTC-3’), miR-668 (Forward primer: 5’-TGTCACTCGGCTCGGC-3’; Reverse primer: 5’-GAACATGTCTGCGTATCTC-3’), and CTLA4 mRNA (Forward primer: 5’-ACGGGACTCTACATCTGCAAGG-3’; Reverse primer: 5’-GGAGGAAGTCAGAATCTGGGCA-3’) in each sample was calculated using the Ct value approach.

### Luciferase assay

To study the molecular relationship between hsa_circ_0003528 and miR-224-5p, miR-324-5p and miR-488-5p, we first constructed wild type luciferase vectors by cloning the sequences of hsa_circ_0003528 into separate pcDNA3.1 vectors (Promega, Madison, WI) between BamHI and HindIII. The correctness of inserted sequence was confirmed by using direct sequencing with common primer: 5' CGCAAATGGGCGGTAGGCGTG 3'. In addition, we used a Quick Change II mutagenesis assay kit (Stratagene, San Diego, CA) in accordance with the standard protocol provided on the product manual of the manufacturer to introduce site directed mutations in the hsa_circ_0003528 binding sites of miR-324-5p, miR-224-5p, and miR-488-5p. In the next step, THP-1 and U937 cells were co-transfected with wild type/mutant type hsa_circ_0003528 using Lipofectamine 2000 together with miR-324-5p, miR-224-5p, and miR-488-5p mimics respectively. At 48 post transfection, the relative luciferase signals of transfected THP-1 and U937 cells were measured on a luminometer using a Dual Luciferase reporter gene assay kit (Promega, Madison, WI) in accordance with the standard protocol provided on the product manual of the manufacturer. Similarly, to study the molecular relationship between CTLA4 and miR-324-5p, miR-224-5p, and miR-488-5p, we first constructed wild type luciferase vectors of CTLA4 3’ UTR by cloning the sequences of CTLA4 3’ UTR containing the miR-324-5p, miR-224-5p, and miR-488-5p binding sites into separate pcDNA3.1 vectors. Then, we used the Quick Change II mutagenesis assay kit to introduce site directed mutations in the miR-324-5p, miR-224-5p, and miR-488-5p binding sites of CTLA4 3’ UTR. In the next step, THP-1 and U937 cells were co-transfected with wild type/mutant type CTLA4 3’ UTR vectors along with miR-324-5p, miR-224-5p, and miR-488-5p mimics using Lipofectamine 2000. At 48 post transfection, the relative luciferase signals of transfected THP-1 and U937 cells were measured on a luminometer using the Dual Luciferase reporter gene assay kit.

### Western blot analysis

The collected samples were first lysed in a RIPA lysis buffer (Sigma Aldrich, St. Louis, MO) in accordance with the standard protocol provided on the product manual of the manufacturer to extract total protein content, which was then separated on a 10% SDS-PAGE gel and transferred onto nitrocellulose membranes. After being blocked in 5% skim milk for 1 h, the nitrocellulose membranes were sequentially probed with primary anti-CTLA4 antibodies and HRP-conjugated secondary antibodies (Abcam, Cambridge, MA, used in accordance with the incubation conditions suggested by the manufacturer). After protein band development using an enhanced chemiluminescence reagent (Clearness ECL substrate, Bio-Rad Laboratories, CA), the relative protein expression of CTLA4 in each sample was quantified by utilizing the ImageJ v1.4.9 software.

### ELISA

The levels of CXCL10, CXCL11, Nos2, CCL17, CCL18, CCL22, TNF-α, IL-6, IL-12, and IL-10 in collected clinical samples were assayed by using commercial ELISA kit (Thermo Fisher Scientific, Waltham, MA) in accordance with the standard protocol provided on the product manual of the manufacturer.

### Statistical analysis

The Student’s tests were done using SPSS 20.0 software (IBM, Chicago, IL). P values of < 0.05 were deemed statistically significant.
